# Awareness of symptoms, anticipated barriers and delays to help-seeking among women at higher risk of breast cancer: A UK multicentre study

**DOI:** 10.1016/j.pmedr.2023.102220

**Published:** 2023-04-25

**Authors:** Sophie M.C. Green, Kelly E. Lloyd, Samuel G. Smith, on behalf of ENGAGE investigators

**Affiliations:** Leeds Institute of Health Sciences, University of Leeds, LS2 9LU, UK

**Keywords:** Breast cancer, Symptom awareness, Help-seeking barriers, Early diagnosis, Familial cancer

## Abstract

Women with a family history of breast cancer have an increased lifetime risk of the disease. Delay in symptom presentation can lead to poorer outcomes. Low awareness of breast cancer symptoms and help-seeking barriers have been associated with delay in presentation in the general population. Symptom awareness and help-seeking barriers among women at increased risk of breast cancer are unknown. We conducted analysis of survey data which included women with moderate and high risk of breast cancer from 20 secondary and tertiary care clinics in England (n = 408). Women completed a validated survey assessing breast cancer symptom awareness, barriers to help-seeking and anticipated delay in help-seeking. Women recognised an average of 9.1/11 breast cancer symptoms (SD = 2.1). Nipple rash was the least recognised symptom (51.0%). Women educated to at least degree level had higher awareness than those with lower education (β = 0.14, 95% CI 0.13, 0.99, *p* = 0.011). Women at lower socioeconomic status (SES) had lower awareness than those at higher SES (β = -0.13, 95% CI −1.09, −0.07, *p* = 0.027). Women reported several anticipated help-seeking barriers (mean = 4.0/11, SD = 2.8). Waiting to see if a symptom will pass was the most commonly reported barrier to help-seeking (71.5%). Most women (376/408; 92.2%) reported that they would seek medical help within 2 weeks of discovering a breast cancer symptom. Interventions to increase awareness of non-lump breast cancer symptoms and reduce help-seeking barriers are needed, with considerations of appropriate reading levels and modalities for women with lower education and SES.

## Highlights

• There was poorer recognition of non-lump symptoms among women at higher breast cancer risk.
• Women with more education had increased awareness of breast cancer symptoms.
• Women who were more deprived recognised fewer symptoms.
• Women at higher risk of breast cancer anticipated barriers to seeking help for symptoms.

## 1 Introduction

Breast cancer is the most common cause of cancer death in women worldwide, and incidence is rising (Bray et al., 2018, Lin et al., 2019). More advanced disease at diagnosis results in worse outcomes including increased mortality (Neal et al., 2015). As the majority of breast cancers are diagnosed following symptomatic presentation to a doctor (Elliss-Brookes et al., 2012, Walker et al., 2014), early diagnosis through prompt reporting of potential symptoms to primary care is a key factor that could improve outcomes, if further diagnostic and treatment steps were taken (Whitaker, 2020).

According to the Model of Pathways to Treatment (Scott et al., 2013, Walter et al., 2012), there are four main intervals between discovery of a symptom and treatment onset. These are (1) appraisal; the time between detecting a bodily change to deciding to discuss this with a medical professional; (2) help-seeking interval; the time from deciding to seek medical help to the first consultation with a health care professional; (3) diagnostic interval; the time between the first appointment and a diagnosis being made; and (4) treatment interval, which is the time from the start of treatment to completion of treatment. Patient factors (e.g. socio-demographics), health system factors and disease related factors can influence each of these intervals.

Increased time taken to present a potential breast cancer symptom to primary care (appraisal interval) may increase the risk of later stage diagnosis (Scott et al., 2013, O'Mahony et al., 2013, Richards, 2009). Limited awareness of breast cancer symptoms can increase this appraisal interval (Moodley et al., 2018). Low awareness of cancer symptoms among the UK general population has been associated with increased time to seek help across a range of cancers, including breast cancer (Robb et al., 2009, Quaife et al., 2014, Khakbazan et al., 2014). Awareness of cancer symptoms in the UK general population is mixed (Robb et al., 2009, Quaife et al., 2014, Waller et al., 2009, Forbes et al., 2011), with knowledge of non-lump breast cancer symptoms (e.g. nipple rash) being particularly poor (Forbes et al., 2011, Ramirez et al., 1999). Across multiple systematic reviews, low awareness of non-lump breast cancer symptoms has been associated with increased delay in seeking medical help (Ramirez et al., 1999, Grimley et al., 2020).

Women with a family history of breast cancer are at increased risk of developing the disease (Nelson et al., 2012). Healthcare professionals can carry out formal risk assessments to estimate patient’s individual risk of developing breast cancer risk. In England, the National Institute for Health and Care Excellence (NICE) classifies women with a lifetime risk of 17–30% as moderate risk of breast cancer, and those exceeding 30% as high risk (National Institute for Health and Care Excellence, 2017). Family history of breast cancer has been associated with increased awareness of breast cancer symptoms among Iranian and Chinese women (Tazhibi and Feizi, 2014, Liu et al., 2014). However, symptom awareness among UK women classified as at higher risk of familial breast cancer is unknown.

Knowledge of cancer symptoms is likely to be one of several factors that affect help-seeking. During the help-seeking interval in the Model of Pathways to Treatment, several barriers to help-seeking may occur (Walter et al., 2012). In the general population, practical barriers to help-seeking include having other things to worry about, and service barriers include finding it difficult to make an appointment (Robb et al., 2009, Waller et al., 2009, Forbes et al., 2011, Heath et al., 2019, Marcu et al., 2017). Emotional barriers impacting help-seeking include worrying what might be found, and feeling too scared or embarrassed (Robb et al., 2009, Waller et al., 2009, Forbes et al., 2011, Heath et al., 2019, Marcu et al., 2017). In cross-sectional and interview studies in the UK population, endorsement of more help-seeking barriers was associated with increased anticipated and actual delay in help-seeking (Robb et al., 2009, Waller et al., 2009, Simon et al., 2010).

Symptom awareness and barriers to help-seeking for cancer symptoms can vary between socio-demographic groups (Bish et al., 2005). In UK studies, lower socioeconomic status (SES), lower education levels, older age and being from an ethnic minority group have all been associated with reduced awareness of cancer symptoms (Waller et al., 2009, Marcu et al., 2017, Linsell et al., 2008, Wood and Scanlon, 2005). Women from ethnic minorities endorse more emotional barriers to help-seeking, in particular increased embarrassment among Bangladeshi, Indian and Pakistani women (Waller et al., 2009, Forbes et al., 2011). Members of the UK general population at lower socio-economic status also experience more emotional barriers, both in terms of being worried what a doctor may find, and lacking confidence in talking to the doctor (Robb et al., 2009). Sociodemographic associations with symptom awareness and help-seeking barriers specifically among UK women at higher risk of breast cancer are unknown.

We aimed to explore breast cancer symptom awareness, anticipated barriers and delays in help-seeking among women at increased risk of breast cancer. Our objectives were to (1) assess levels of symptom awareness, anticipated barriers to help-seeking, and anticipated delay in help-seeking among women at higher breast cancer risk and (2) investigate the sociodemographic and clinical factors associated with these outcomes.

## 2 Methods

This analysis is part of the ENGAGE study. The ENGAGE study was a large UK multicentre prospective project, investigating cancer prevention decision-making in women with a family history of breast cancer (Hackett et al., 2018, Thorneloe et al., 2019). The ENGAGE project aimed to prospectively investigate uptake of tamoxifen following the introduction of the 2013 NICE guidelines on familial breast cancer (Hackett et al., 2018, Thorneloe et al., 2019). In the present study, we analysed specific measures from the ENGAGE baseline data, including the validated Breast Cancer Awareness Measure (Breast-CAM) (Linsell et al., 2010, CRUK, 2011), which have not yet been analysed, to investigate awareness of breast cancer symptoms, help-seeking barriers endorsed, and anticipated delay in help-seeking among women at higher risk of breast cancer.

### 2.1 Recruitment

Participants were recruited across 20 secondary and tertiary clinics. These included family history clinics (n = 12), breast clinics (n = 4), clinical genetic centres (n = 3) and a family history clinic with genetics support (n = 1). Most clinics were in major cities across England, and recruitment was open between September 2015 and December 2016. Following an appointment at the clinic, women were approached by a research nurse or healthcare professional to discuss the study. Women who verbally consented were given an ENGAGE study pack, which contained the survey and a freepost envelope. Participants who did not return the survey after 2 weeks were sent a reminder postcard, and after 4 weeks were resent the survey. The study team were sent the personal data of women who verbally consented to participate in the study team via a secure online portal. The patient’s data supplied was their email address, home address, age, and their risk classification (‘moderately high’ or ‘high’).

The ENGAGE study was granted ethical approval by the National Research Ethics Service Committee North West – Preston (14/NW/1408). Women were eligible to participate if they were classified as having moderately high or high risk of developing breast cancer according to NICE guidance CG164 (Hackett et al., 2018) (Full inclusion criteria in Table 1). Women provided verbal consent to their details being passed on to the research team, and then consent was implied following the return of the written questionnaire. Women were excluded if they were unable to read English (as the survey was only available in English), had a previous diagnosis of breast cancer, or the health care professional felt they did not have the mental capacity to consent. All procedures performed in studies involving human participants were in accordance with the ethical standards of the institutional and/or national research committee and with the 1964 Declaration of Helsinki and its later amendments or comparable ethical standards.

**Table 1:** Inclusion and exclusion criteria for women at increased risk of breast cancer recruited to the UK ENGAGE study, 2015–2016.

Inclusion Criteria	Exclusion Criteria
Over 18 years old	Unable to read English
Able to speak English	Had a previous diagnosis of breast cancer
Moderately high, or high risk of breast cancer, according to NICE guidance CG164	Do not have mental capacity for informed consent
Discussed preventive therapy with a healthcare professional	
No known contraindications for Tamoxifen use	

### 2.2 Measures

The following measures from the ENGAGE baseline survey were used for the current analysis.

#### 2.2.1 Socio-demographic and clinical factors

We recorded self-reported education level (‘≥degree level’ vs. ‘<degree level’), ethnicity (‘white ethnic groups’ vs. ‘ethnic minority groups’) and perceived health status (‘poor’, ‘fair’, ‘good’, ‘excellent’). We assessed numeracy with a single item (“Which of the following numbers do you think represents the biggest risk of getting a disease?”), with three response options (e.g. 1 in 100). Numeracy was dichotomised as “poor” if the answer was incorrect, and “good” if the answer was correct. Participants’ breast cancer risk category was provided by staff at the clinic (‘moderately high’ risk or ‘high’ risk of developing breast cancer, from NICE guidance CG164 (Hackett et al., 2018)). Participants’ age was calculated from their National Health Service (NHS) records. To calculate SES, participants’ postcodes were used to calculate their Index of Multiple Deprivation score (McLennan et al., 2011), and were categorised into tertiles of neighbourhood deprivation (‘low’, ‘middle’ and ‘high’). The full survey is available from https://osf.io/mqz9y/.

#### 2.2.2 Signs and symptoms of breast cancer

Eleven items were used to measure recognition of breast cancer symptoms, which were adapted from the validated Breast Cancer Awareness Measure (Breast-CAM) (Linsell et al., 2010, CRUK, 2011). All items were known symptoms of breast cancer. Participants were asked if any of the following items could be a sign of breast cancer. Example items included a lump or thickening in the breast, pain in breast or armpit, and nipple rash. Options for each item included ‘Yes’, ‘No’, or ‘Unsure’. For the purpose of analysis, ‘No’ and ‘Unsure’ were grouped into one variable.

#### 2.2.3 Help-seeking barriers

We measured barriers to help-seeking that were included in the Breast-CAM scale (Linsell et al., 2010, CRUK, 2011). Participants were asked if any of the following items might put them off going to the doctor, with options ‘Yes, often’, ‘Sometimes’, and ‘No’. Items included practical barriers (e.g. ‘Difficult to arrange transport to the doctor’s surgery’), service barriers (e.g. ‘Difficult to make an appointment with the doctor’), and emotional barriers (e.g. ‘Too embarrassed to go and see the doctor’). In addition to the 10 items from the Breast-CAM scale (Linsell et al., 2010, CRUK, 2011), participants were asked if ‘Waiting to see if a symptom will pass on its own’ was a barrier to help-seeking. Responses of ‘Yes, often’ and ‘Sometimes’ were grouped into one variable to calculate an overall number of help-seeking barriers reported per participant.

#### 2.2.4 Anticipated delay to help-seeking

Participants were asked ‘If you found a change in your breasts, how soon would you contact your doctor?’, from the Breast-CAM scale (Linsell et al., 2010, CRUK, 2011). Options included ‘Immediately’, ‘1–2 days’, ‘Within a week’, ‘Within 2 weeks’, ‘Within a month’, ‘Within 3 months’, and ‘Never’. Anticipated delay responses were recoded to ‘≤2 weeks’ and ‘>2 weeks’, as used in previous analyses (Robb et al., 2009, Quaife et al., 2014).

### 2.3 Analysis

We reported levels of breast cancer symptom awareness, number of help-seeking barriers endorsed, and anticipated delay in help-seeking in proportions, frequencies, and means. Univariable and multivariable linear regression models were conducted to investigate the relationship between participants’ socio-demographic and clinical factors on the outcomes of number of breast cancer symptoms identified and help-seeking barriers endorsed. We intended to use univariable and multivariable logistic regression models to examine whether awareness of breast cancer symptoms and number of help-seeking barriers endorsed were associated with an anticipated delay in help-seeking (‘≤2 weeks’ and ‘>2 weeks’). However, we did not complete these analyses due to insufficient numbers in the ‘delay to help-seeking’ group. All analyses were conducted in SPSS version 26, with statistical significance set at a 2-sided *p* < 0.05.

## 3 Results

### 3.1 Demographics

In total, 732 women were invited to complete the survey, and 408 (55.7%) completed the survey. There were no significant differences in age (*p* = 0.086), clinical risk (*p* = 0.62), or SES (*p* = 0.054) between survey responders (408 women) and non-responders (324 women). Participant characteristics are displayed in Table 2. The mean age of participants was 45.30 (SD 7.82), with most participants falling within the 36–49 age bracket (63.5%). Most women were from white ethnic groups (384/408; 94.1%), had children (314/408; 77.0%), were married (298/408; 73.0%) and were employed full-time (348/408; 85.3%). Most participants reported a good health status (240/408; 58.8%) and had good numeracy (318/408; 77.9%). Most participants were classified as moderate risk of breast cancer (243/408; 59.6%).

**Table 2:** Demographic and clinical characteristics for women at increased risk of breast cancer recruited to the UK ENGAGE study, 2015–2016 (n = 408).

Demographics			N (%)
Age, mean (SD)			45.3 (7.82)

Age, n (%)			
	≤35 years		41(10.0)
	36–49 years		259 (63.5)
	≥50 years		108 (26.5)

Children, n (%)			
	Yes		314 (77.0)
	No		94 (23.0)

Ethnicity, n (%)			
	White ethnic groups		384 (94.1)
	Ethnic minority groups		18 (4.4)
		Caribbean	3
		Indian	5
		Pakistani	2
		Other Asian	1
		White and Black Caribbean	2
		White and Black African	1
		Other mixed ethnicity	2
		Any other	2
	Missing		6 (1.5)

Education level, n (%)			
	Degree or above		176 (43.1)
	Below degree level		222 (54.4)
	Missing		10 (2.5)

Employment Status, n (%)			
	Full time		348 (85.3)
	All other employments		60 (14.7)

Marital Status, n (%)			
	Married/ cohabiting		298 (73.0)
	Unmarried		103 (25.2)
	Missing		7 (1.7)

Health status, n (%)			
	Excellent		66 (16.2)
	Good		240 (58.8)
	Fair		78 (19.1)
	Poor		16 (3.9)
	Missing		8 (2.0)

SES, n (%)			
	Low		120 (29.4)
	Medium		131 (32.1)
	High		150 (36.8)
	Missing		7 (1.7)

Numeracy, n (%)			
	Good		318 (77.9)
	Poor		72 (17.6)
	Missing		18 (4.4)

Risk level, n (%)			
	Moderate		243 (59.6)
	High		159 (39.0)
	Unclear		6 (1.5)

Key: SD = Standard deviation. SES = Socioeconomic status.

### 3.2 Symptom awareness

**Fig. 1:**
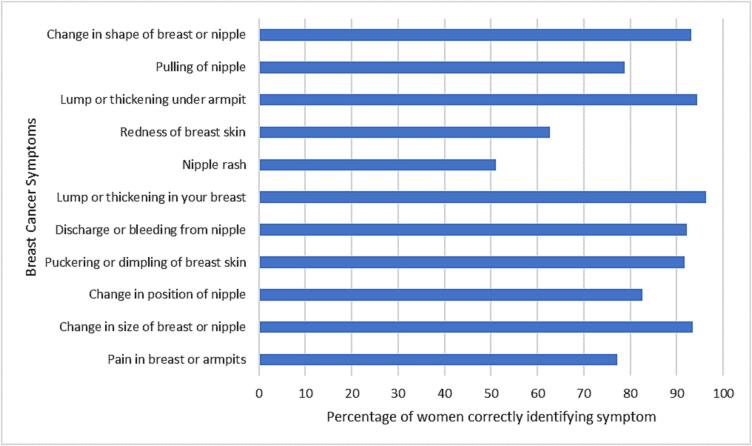
Recognition of breast cancer symptoms among women at increased risk of breast cancer in the UK ENGAGE study, 2015–2016 (n = 408).

Out of 11 breast cancer symptoms presented, the mean number of symptoms recognised was 9.1 (SD 2.1) (Fig. 1, Appendix Table A.1). Lump or thickening in your breast was recognised by the most women (393/408; 96.3%). Nipple rash was recognised as a symptom by the fewest number of women (208/408; 51.0%), followed by redness of breast skin (256/408; 62.7%).

A multivariable linear regression model examined the relationship between the number of symptoms women are aware of and their socio-demographic and clinical variables. In the multivariable model (n = 384), those educated at degree level or above (vs. less than degree level) were aware of a higher number of breast cancer symptoms (β = 0.14, 95% CI 0.13, 0.99, *p* = 0.011; Table 3). Those from a lower SES background (vs. high SES; β = -0.13, 95% CI −1.09, −0.07, *p* = 0.027) and those classified as having an unclear level of risk (vs. moderate risk; β = -0.11, 95% CI −3.49, −0.22, *p* = 0.027) were aware of fewer breast cancer symptoms. In univariable models, those with good numeracy were aware of fewer symptoms of breast cancer (vs. poor numeracy; β = -0.15, 95% CI 0.09, 1.13, *p* = 0.007), but this did not retain significance in the multivariable model.

**Table 3:** Multiple linear regression to explore socio-demographic and clinical factors associated with number of breast cancer symptoms recognised, among women at increased risk of breast cancer in the UK ENGAGE study, 2015–2016 (n = 384).

			Univariable		Multivariable	
Symptom Awareness, mean (SD)			β (95% CI)	*P* value	β (95% CI)	*P* value
Age		-	−0.05(−0.04, 0.01)	0.330	0.01(−0.03, 0.03)	0.872

Education						
	Degree level and above	9.6(1.8)	0.16 (0.27, 1.07)	**0.001**	0.14 (0.13, 0.99)	**0.011**

	Below degree level	8.9(2.2)	Ref.		Ref.	

Ethnicity						
	White	9.2(2.0)	Ref.		Ref.	
	Ethnic minority	8.9(2.4)	−0.03(−1.21, 0.71)	0.609	0.00 (−0.99, 1.08)	0.939

Health Status						
	Poor	8.6(2.3)	−0.08(−2.07, 0.25)	0.122	−0.07 (−1.80, 0.45)	0.237
	Fair	8.9(2.3)	−0.10(−1.22, 0.14)	0.122	−0.08 (−1.07, 0.30)	0.268
	Good	9.1(2.0)	−0.08(−0.90, 0.21)	0.226	−0.08 (−0.91, 0.22)	0.228
	Excellent	9.7(1.7)	Ref.		Ref.	

SES						
	High	9.4(1.8)	Ref.		Ref.	
	Medium	9.3(2.2)	−0.02(−0.56, 0.39)	0.684	−0.05 (−0.69, 0.30)	0.430
	Low	8.7(2.2)	−0.15(−1.20, −0.19)	**0.007**	−0.13 (−1.09, −0.07)	**0.027**

Numeracy						
	Poor	8.7(2.4)	Ref.		Ref.	
	Good	9.3(2.0)	0.12(0.09, 1.13)	**0.022**	0.09 (−0.07, 0.99)	0.092

Risk Level						
	Moderate	9.2(2.1)	Ref.		Ref.	
	High	9.2(2.1)	0.00(−0.43, 0.42)	0.973	0.01 (−0.38, 0.46)	0.855
	Unclear	7.2(3.5)	−0.11(−3.73, −0.27)	**0.024**	−0.11 (−3.49, −0.22)	**0.027**

Bold text indicates statistical significance (*P* ≤ 0.05).

Key: SD = Standard deviation. CI = Confidence interval. Ref = Reference category. SES = Socioeconomic status.

### 3.3 Barriers to help-seeking

**Fig. 2:**
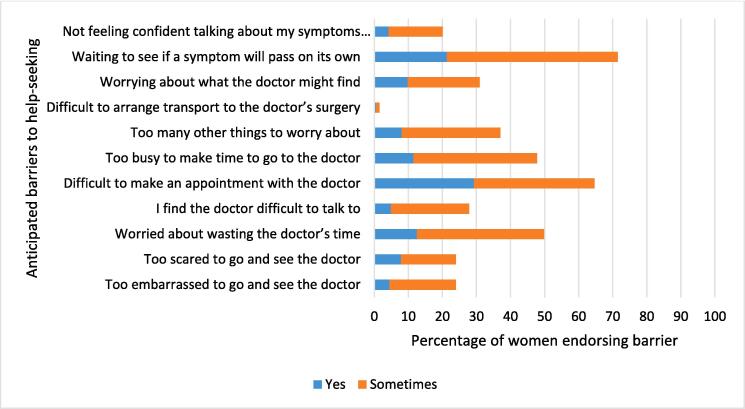
Reported anticipated barriers to help-seeking upon discovering a symptom of breast cancer, among women at increased risk of breast cancer in the UK ENGAGE study, 2015–2016 (n = 408).

The mean number of barriers to help-seeking reported was 4.0 (SD 2.8), out of a possible 11 barriers. Waiting to see if a symptom will pass on its own was the most frequently reported barrier (292/408; 71.5% reported ‘yes’ or ‘sometimes’), followed by difficulty making an appointment with the doctor (263/408; 64.5% reported ‘yes’ or ‘sometimes’) (Fig. 2, Appendix Table A.2). Difficulty arranging transport to the doctor’s surgery was experienced by a small number of women (6/408; 1.5% reported ‘yes’ or ‘sometimes’).

In univariable linear regression models examining the association between the number of barriers to help-seeking and socio-demographic and clinical variables, poor (β = 0.11, 95% CI 0.07, 3.03, *p* = 0.040), fair (β = 0.16, 95% CI 0.24, 1.98, *p* = 0.013) and good health status (β = 0.16, 95 %CI 0.16, 1.59, *p* = 0.016) were all associated with an increased number of barriers to help-seeking compared to those reporting excellent health (Table 4). In the multivariable linear regression model, including all socio-demographic and clinical factors, none of these groups remained significant. No other socio-demographic or clinical factors were associated with the number of reported barriers to help-seeking in the multivariable model.

**Table 4:** Multiple linear regression to explore socio-demographic and clinical factors associated with barriers to help seeking, among women at increased risk of breast cancer in the UK ENGAGE study, 2015–2016 (n = 384).

			Univariable		Multivariable	
Barriers reported, mean (SD)			β (95% CI)	*P* value	β (95% CI)	*P* value
Age		–	−0.07(−0.06, 0.01)	0.151	−0.07(−0.06, 0.01)	0.197

Education						
	Degree level and above	4.3(2.6)	0.09(−0.07, 1.02)	0.088	0.07(−0.19, 0.99)	0.186
	Below degree level	3.8(2.8)	Ref.		Ref.	

Ethnicity						
	White	4.0(2.8)	Ref.		Ref.	
	Ethnic minority	4.1(2.3)	0.01(−1.22, 1.38)	0.901	−0.01(−1.57, 1.28)	0.844

Health Status						
	Poor	4.8(2.8)	0.11(0.07, 3.03)	**0.040**	0.10(−0.25, 2.84)	0.100
	Fair	4.3(3.0)	0.16(0.24, 1.98)	**0.013**	0.13(−0.08, 1.80)	0.072
	Good	4.1(2.6)	0.16(0.16, 1.59)	**0.016**	0.13(−0.04, 1.50)	0.063
	Excellent	3.4(2.8)	Ref.		Ref.	

SES						
	High	3.8(2.9)	Ref.		Ref.	
	Medium	4.1(2.7)	0.04(−0.38, 0.90)	0.429	0.03(−0.53, 0.82)	0.676
	Low	4.2(2.7)	0.06(−0.28, 1.03)	0.256	0.03(−0.51, 0.90)	0.585

Numeracy						
	Poor	4.1(2.8)	Ref.		Ref.	
	Good	4.0(2.7)	−0.02(−0.81, 0.59)	0.763	−0.02(−0.88, 0.59)	0.701

Risk Level						
	Moderate	4.0(2.7)	Ref.		Ref.	
	High	4.0(2.8)	0.00(−0.54, 0.56)	0.976	−0.00(−0.58, 0.58)	0.999
	Unclear	2.8(2.1)	−0.05(−3.40, 1.06)	0.303	−0.06(−3.59, 0.91)	0.243

Bold text indicates statistical significance (*P* ≤ 0.05).

Key: SD = Standard deviation. CI = Confidence interval. Ref. = Reference category. SES = Socioeconomic status.

### 3.4 Anticipated delay in help-seeking

The majority of women in the sample were not classified as delayed in help-seeking, reporting that they would seek medical help within 2 weeks of discovering a potential breast cancer symptom (376/408; 92.2%) (Table 5). Of the 6.9% (28/408) of women who reported anticipated delay in help-seeking, 6.4% (26/408) reported seeking medical help within a month, while 0.5% (2/408) reported seeking help within 3 months. Univariable and multivariable models exploring predictors of anticipated help-seeking were not conducted due to the small sample size within the delayed help-seeking group.

**Table 5:** Reported anticipated delay to help seeking upon discovering a symptom of breast cancer, among women at increased risk of breast cancer in the UK ENGAGE study, 2015–2016 (n = 408).

Anticipated delay in help seeking		N (%)

Not delayed		376 (92.2)
	Immediately	183 (44.9)
	1–2 days	69 (16.9)
	Within a week	89 (21.8)
	Within 2 weeks	35 (8.6)

Delayed		28 (6.9)
	Within a month	26 (6.4)
	Within 3 months	2 (0.5)

Missing		4 (1.0)

## 4 Discussion

In this UK study involving women at higher risk of breast cancer, there was good recognition of most breast cancer symptoms, but poorer recognition for non-lump symptoms such as nipple rash and skin redness. Despite participants being at increased risk of breast cancer, several anticipated barriers to seeking medical help for a cancer symptom were highly endorsed, such as waiting for a potential symptom to pass before contacting their doctor. However, most women reported they would seek medical help after identifying a potential breast cancer symptom within 2 weeks.

Recognition of lump-based breast cancer symptoms in our sample of women at higher risk of breast cancer was similar to the general population. In our sample, 96.3% and 94% of women recognised a lump in their breast and armpit respectively as a symptom of breast cancer, similar to findings in two UK based population studies exploring cancer awareness in the general population (Robb et al., 2009, Quaife et al., 2014). In our sample, non-lump symptoms such as nipple rash and redness of breast skin were recognised by 51% and 62.7% of women respectively. This reflects previous findings identifying that non-lump based symptoms are less well recognised in general and breast-cancer specific population studies (Robb et al., 2009, Quaife et al., 2014, Forbes et al., 2011, Ramirez et al., 1999, Grimley et al., 2020). Targeted support to increase awareness of non-lump breast cancer symptoms among both the general population and those at higher risk of the disease is warranted.

Our study found evidence for existing health inequalities affecting symptom awareness among women at higher cancer risk, with those at lower SES and lower levels of education presenting with poorer symptom recognition. These sociodemographic associations reflect findings in general population samples (Robb et al., 2009, Quaife et al., 2014, Ramirez et al., 1999, Marcu et al., 2017). Women with lower SES and a lower level of education may not interpret symptoms as worrying, and may be less likely to associate symptoms with cancer, increasing the appraisal interval (Scott et al., 2013, Robb et al., 2009, Marcu et al., 2017, Linsell et al., 2008). In a study assessing awareness of gynaecological symptoms, participants with lower health literacy benefited significantly less from an educational leaflet intervention than those with higher health literacy (Boxell et al., 2012). This indicates the importance of targeted support for women at lower SES and education level.

In our sample, practical and service barriers were endorsed more than emotional barriers to help-seeking. Practical and service barriers most commonly endorsed in previous UK based population studies include difficulty making an appointment with the doctor, and not wanting to waste the doctor’s time. These barriers were similarly frequently endorsed within our sample (Robb et al., 2009, Forbes et al., 2011), in which worry about what a doctor may find was the most endorsed emotional barrier to help-seeking (30.9%). These barriers are likely to increase the help-seeking interval in the model of pathways to treatment (Scott et al., 2013). Emotional barriers to help-seeking may be more amenable to change than practical and service level barriers, and therefore offer a potential intervention target aimed at reducing delays in presentation.

### 4.1 Implications for practice

There is a need for targeted support to increase awareness of several non-lump based breast cancer symptoms and reduce help-seeking barriers among women at higher risk of the disease. Previous interventions have successfully improved cancer symptom recognition among the general population. For example, the Promoting Early Presentation intervention involved a scripted interaction between a radiographer covering breast cancer symptoms and how to check for breast changes, supplemented by an educational booklet. The intervention increased breast cancer symptom awareness in older women, but had limited effects on help-seeking barriers (Forbes et al., 2011, Forbes et al., 2012). An educational intervention based on the Health Belief Model, which included topics on breast cancer symptoms, also increased breast cancer symptom awareness among women from the general public (Torbaghan et al., 2014). There is scope for similar interventions to be developed specifically for women at increased risk of breast cancer. Interventions should focus on increased education about non-lump based symptoms, and strategies to reduce emotional barriers to help-seeking that are amenable to change. In the development of any new intervention, there should be specific consideration of the readability and literacy of any written materials, preferences of women in these socioeconomic groups, and the use of visual materials, such as pictures or videos that could be beneficial for those with lower education levels (Sheridan et al., 2011, Mbanda et al., 2021).

### 4.2 Limitations

The study had limitations. We collected data on recognition of a list of presented symptoms, however previous evidence has found symptom recall is significantly lower when using an unprompted recall format (Waller et al., 2004). Therefore, our study may have overestimated awareness of symptoms. Participants reported hypothetical barriers and delay to seeking medical help for cancer symptoms; actual experienced barriers and delay may be different in clinical practice. Only 18 participants were recruited from an ethnic minority background, which reduces confidence in our finding of no association between ethnicity and the study outcomes. As ethnic minority groups in the general population have been found to report lower symptom awareness and higher endorsement of help-seeking barriers, further research investigating these outcomes among ethnic minorities at higher cancer risk is warranted (Waller et al., 2009, Wood and Scanlon, 2005). Reponses to the survey may have been hindered by selection bias, as over 40% of women who consented to participate in the study did not return the survey. The data were collected between 2015 and 2016, and therefore do not reflect barriers to help-seeking that may have been exacerbated during the coronavirus pandemic (Borsky et al., 2022, Quinn-Scoggins et al., 2021).

### 4.3 Conclusion

In this UK study involving women at higher risk of breast cancer, there was poorer recognition of non-lump symptoms, and several help-seeking barriers were endorsed. Lower education and SES were associated with reduced symptom awareness, indicating a need for interventions to support this group. Increasing symptom awareness, particularly of non-lump based symptoms of breast cancer, and reducing help-seeking barriers could reduce time from symptom identification to presentation, and consequently improve outcomes among women at higher risk of breast cancer.

### CRediT authorship contribution statement

**Sophie M.C. Green:** Formal analysis, Conceptualization, Data curation, Writing – original draft, Writing – review & editing. **Kelly E. Lloyd:** Formal analysis, Conceptualization, Data curation, Writing – original draft, Writing – review & editing. **Samuel G. Smith:** Funding acquisition, Conceptualization, Methodology, Data curation, Writing – review & editing, Supervision, Project administration.

## Declaration of Competing Interest

The authors declare that they have no known competing financial interests or personal relationships that could have appeared to influence the work reported in this paper.

## Figures and Tables

**Table A.1:** Recognition of breast cancer symptoms (n = 408).^a^

Symptom	Yes n(%)	No/Unsure n(%)
Pain in breast or armpits	315 (77.2)	90 (22.1)
Change in size of breast or nipple	381 (93.4)	25 (6.1)
Change in position of nipple	337 (82.6)	68 (16.6)
Puckering or dimpling of breast skin	374 (91.7)	32 (7.8)
Discharge or bleeding from nipple	376 (92.2)	30 (7.3)
Lump or thickening in your breast	393 (96.3)	13 (3.3)
Nipple rash	208 (51.0)	198 (48.5)
Redness of breast skin	256 (62.7)	150 (36.7)
Lump or thickening under armpit	385 (94.4)	21 (5.2)
Pulling of nipple	321 (78.7)	85 (20.8)
Change in shape of breast or nipple	380 (93.1)	25 (6.1)

^a^Missing data ranged from 2 to 3 participants per symptom (0.5-0.7%).

**Table A.2:** Anticipated barriers to help seeking (n = 408).^a^

Barrier to help seeking	Yes, n (%)	Sometimes, n (%)	No, n (%)
Too embarrassed to go and see the doctor	18(4.4)	80(19.6)	307(75.2)
Too scared to go and see the doctor	32(7.8)	66(16.2)	306(75.0)
Worried about wasting the doctor’s time	51(12.5)	152(37.3)	202(49.5)
I find the doctor difficult to talk to	20(4.9)	94(23.0)	290(71.1)
Difficult to make an appointment with the doctor	119(29.2)	144(35.3)	142(34.8)
Too busy to make time to go to the doctor	47(11.5)	148(36.3)	209(51.2)
Too many other things to worry about	33(8.1)	118(28.9)	252(61.8)
Difficult to arrange transport to the doctor’s surgery	2(0.5)	4(1.0)	397(97.3)
Worrying about what the doctor might find	40(9.8)	86(21.1)	278(68.1)
Waiting to see if a symptom will pass on its own	87(21.3)	205(50.2)	113(27.7)
Not feeling confident talking about my symptoms with the doctor	17(4.2)	65(15.9)	323(79.2)

^a^Missing data ranged from 3 to 5 participants per barrier (0.7–1.2%).

## Data Availability

Data will be made available on request.

## Appendix A

Tables presenting the number and proportion of women who recognised each breast cancer symptom and endorsed each help-seeking barrier.
